# Evidence-based practice implementation and associated factors among nurses employed at public hospitals in Siltie Zone, South Central Ethiopia: A cross-sectional study

**DOI:** 10.1371/journal.pone.0357626

**Published:** 2026-10-20

**Authors:** Sadik Seid Ahmed, Altayework Mekonnen Gebresillassie, Tigist Tsegaye Bekele, Habtamu Ayalew Mekonnen

**Affiliations:** Saint Paul’s Hospital Millennium Medical College School of Nursing Department of Medical-Surgical Nursing, Addis Ababa, Ethiopia; Karolinska Institutet, SWEDEN

## Abstract

**Background:**

The use of the best available research evidence in clinical decision-making through the incorporation of clinical experience and patient preferences into the delivery of professional patient care is known as evidence-based practice. The best way to provide high-quality care that is secure and has positive results is through evidence-based practice. To use evidence-based practice. Despite the anticipated advantages of evidence-based practice, nurses have found it challenging to implement.

**Objectives:**

To assess the implementation of evidence-based practice and related factors among nurses working in public hospitals in the Siltie Zone in South Central Ethiopia in 2023.

**Methods:**

An institution-based cross-sectional study was conducted from March 6 to April 5, 2023, to assess the implementation of evidence-based practice. A sample size was 337 nurses working in Siltie Zone public hospitals; a self-administered, semi-structured questionnaire was used for data collection. Data was cleaned, coded, and entered into EpiData version 4.6 before being exported and analyzed in SPSS (version 26). To ascertain the relationship between the outcome variable and the predictors, binary logistic regression was performed, and those with a p-value of < 0.25 were entered into multivariate analysis. In multivariate analysis, a p-value < 0.05 was considered statistically significant. The Adjusted Odds Ratio (AOR) was calculated to determine the association between the dependent and independent variables at a 95% confidence interval (CI).

**Result:**

According to this study, 147 (45.5%) of nurses have good evidence-based practice implementation, and 176 (54.5%) of nurses were found to have poor evidence-based practice implementation. Factors significantly associated with implementation of evidence-based practice were age between 26 and 30 (AOR = 5.247, 95% CI: 1.249, 22.047); educational level, BSc (AOR = 1.956, 95% CI: 1.035, 3.698) and masters (AOR = 4.149, 95% CI: 1.088, 15.817); working unit like emergency (AOR = 3.935, 95% CI: 1.300, 11.914) and inpatient (AOR = 4.684, 95% CI: 1.633, 13.435); ability to formulate a question (AOR = 1.844, 95% CI: 1.062, 3.201); and not relevant to the nursing profession (AOR = 0.459, 95% CI:  .236,  .878).

**Conclusion:**

This study showed that the majority of nurses implement evidence-based practice ineffectively. Age, educational level, working unit, question formulation, and the applicability of evidence-based practice to nursing professions were the main causes of inadequate implementation of evidence-based practice. Therefore, multi-stakeholder approaches are required to address the main contributing variables and promote the implementation of evidence-based practice.

## Background

Evidence-based practice has long been a priority on a global scale. The European Commission and the World Health Organization both stress that social and health services should be based on the most recent and reliable scientific evidence [1]. Evidence-based care is the delivery of expert patient care that incorporates the best available research evidence into clinical decision-making while also taking into account patient preferences and clinical experience. Nursing is a science that bases its clinical judgments on the most recent and top-quality research information. Nursing practice generates research questions, yet current practice is founded on research [2].

EBP in healthcare seeks to provide quality patient care by using the most recent, reliable scientific data [3]. EBP is sometimes referred to as a process that involves several steps, including formulating research questions, locating and compiling information, employing the information, and evaluating the practice that follows [4]. When nursing treatment is based on the greatest available scientific information, patient outcomes have been seen to improve.

Over 50% of all health professionals work in nursing, which is the foundation of the majority of health-care systems worldwide. It was discovered that 32.1% of clinicians hardly, rarely, or never looked for research to support their clinical practice [5]. Only 5% of patients were treated using evidence-based therapy, according to a study in China, and 2% were treated using evidence-based practice [6]. To effectively utilize EBP, the nursing profession is crucial [7]. According to some experts, tradition, intuition, and personal experience have a greater influence on nursing care than scientific study. Numerous studies from different countries have shown that nurses rarely perform particular EBP duties [8].

The major contributing factors to implementing EBP were found to be the work institution (setting), the individuals (nurses), and the presentation of research findings [9]. According to Pickler (2018), nursing science needs to incorporate all sorts of research, from discovery to translation, from bench to bedside, and from mechanistic to holistic [10]. To guarantee high-quality care, patient safety, and improved patient outcomes, evidence must be translated into practice [11]. A problem-solving approach used in the delivery of healthcare called evidence-based practice (EBP) combines the best data from studies and patient care records with the expertise of the doctor caring for the patient, as well as their preferences and values. The highest quality of treatment and the best patient outcomes can be provided when they are done in an environment of compassion and in an encouraging corporate culture [12]. EBP also shortens hospital stays and lowers their costs. Evidence-based practice is preferred over the conventional nursing approach for enhancing abilities in the nursing profession because it fosters the development of critical thinking and creativity [13].

Despite the significance of clinical judgments, little is known about the kinds of uncertainties that healthcare professionals, particularly nurses, encounter. Examining the clinical questions that nurses ask when making clinical choices is one method of conveying this uncertainty and the information needs that follow from them [14]. Nurses must take into account the patient’s preferences and likely actions in terms of what interventions she or he is ready and able to accept [15]. The most important factor in clinical practice is patient preference [16]. The EBP procedure is fundamentally based on patient preferences [17].

EBP is being advocated for nurses. EBP is also being emphasized in Africa, but Africa lags in research as fewer nurses are available who are sufficiently prepared at a higher level to enable them to conduct research because of the few higher institutions and the exodus of nurses to developed countries for greener pastures. Lack of funding is also another obstacle for the nurses to conduct research, regardless of having research knowledge [18]. The federal Ministry of Health in Ethiopia lacks skilled medical professionals who could assist in the analysis of data for policymaking. Additionally, there is little tradition or culture at any level of the health systems for accepting or using evidence [19].

According to some researchers, nurses lack understanding of EBP and have a negative attitude toward it. Many nurses are not familiar with the meaning of the term “EBP” or how to use it in clinical practice [20]. Therefore, this study is aimed at assessing evidence-based practice implementation and associated factors among nurses working in public hospitals in the Siltie Zone in South Central Ethiopia, which is significant for nurses and healthcare professionals to provide the highest quality of care in meeting the needs of patients and families as a whole and to decrease health care costs, which is a priority of governmental and funding agencies.

### Rational of the study

This research is aimed to assess EBP among nurses working in government hospital in Siltie zone. Since the Siltie zone is one of the country’s rural settings where transportation and electric supply are limited, nurses are supposed to encounter some difficulty carrying out EBP. Hence, this study’s finding will help to give some important findings regarding EBP of nurses in other rural settings of the country in the same condition.

### General objective

To assess evidence-based practice implementation and associated factors among nurses working in public hospitals in the Siltie Zone in South Central Ethiopia. 2023

### Specific objectives

To determine evidence-based practice implementation among nurses working in Siltie Zone public hospitals in South Central Ethiopia, 2023.

To identify factors associated with EBP implementation among nurses working in Siltie Zone public hospitals, south central Ethiopia, 2023.

## Methods

### Study area and period

The study was conducted at Siltie Zone public hospitals from March 6 to April 5, 2023. The Siltie zone is found in SNNPR (South Nations, Nationalities, and Peoples’ Region), and it has its own language, ethnicity, and culture. The Siltie zone is located 172 km south of Addis Ababa, the capital of Ethiopia, and 202 km from Hawassa, the capital of the South Regional State. In the 2010 Ethiopian Calendar (E.C.) population projection, the Siltie zone consists of a total population of about 1,007,660, with males 498,792 (49.5%) and females 508,868 (50.5%). According to the health department’s health profile for the Siltie Zone, there is one comprehensive specialty hospital and three primary hospitals in the area. Worabe Comprehensive Specialized Hospital was inaugurated on November 29, 2014, and is the first comprehensive specialized hospital in the nation. The hospital is staffed with a total of 1028 health professionals, of whom 207 are nurses. There are three primary governmental hospitals in the Siltie zone. These are the Kibet Primary Hospital, Tora Primary Hospital, and Alemgebeya Primary Hospital, which are administered under the siltie zone health departments. This study is focused on these three primary hospitals in the siltie zone and one comprehensive specialized hospital found in the siltie zone.

### Study design

An institution-based cross-sectional study design was employed among nurses at the Siltie Zone public hospitals from March to April 2023

### Study population

The study population was all nurses working in the Public Hospitals of Siltie Zone from March 6-April 5/2023.

### Inclusion criteria

All nurses were included in the study.

### Exclusion criteria

Nurses who are students, involuntarily absent or unavailable during the study period, were excluded.

### Sample size determination

A Census of all eligible nurses (N=337) was conducted

### Sampling procedure

All nurses were sampled.

### Instruments

For data gathering, a semi-structured questionnaire was used. A self-administered, semi-structured questionnaire that had been previously evaluated and was based on several pieces of literature was used to gather the data [5,7,18,21–24]. The questionnaires are prepared in English and composed of six major portions: socio-demographic, knowledge, attitude, practice, implementation of EBP, and aspects associated with evidence-based practice. As data collectors and supervisors, three BSc nurses and one MSc nurse, respectively, were hired. On how to facilitate the data collection procedure, about the purpose of the study, confidentiality, and verbal consent, they received a one-day training.

### Study variable

### Dependent variables

Evidence-based practice and implementation

### Independent variable

#### Socio-demographic variables.

Age, gender, marital status, educational level, work experience, position, working units, and working hours).

**Individual variables** (knowledge, attitude, and practice of EBP, time shortage, unwillingness to new idea, evaluating research, formulating questions, lack of ability to use a computer, lack of awareness of research articles and relevance to nursing).

**Institutional variables** (work overload, shortage of nurses, insufficient time, lack of internet access, lack of computer, lack of access to reach the library, lack of administrative support, and lack of training).

**Data collection and procedure:** The data were collected using a semi-structured self-administered questionnaire to evaluate the nurse’s EBP implementation and to identify associated factors. The questionnaires were written in English because the study participants were nurses who were thought to be fluent in English. The Principal Investigator (PI) did continuous monitoring and supervision throughout the data collection period.

### Operational definitions

**Sufficient knowledge**: respondents who answered correctly and those who scored equal to or above the overall mean value from the total of knowledge-related questions [22].

**Insufficient knowledge**: respondents who answered below the overall mean value from the total of knowledge-related questions [22].

**Attitude**: how nurses perceive (beliefs) the importance of EBP in their clinical setting.5-point Likert scale, which ranges from five or more answers agree or strongly agree, has a positive perception from nine questionnaires [18].

**Positive attitude**: respondents who answered agree or strongly agree, with a mean of forty-five and above from forty-five Likert scale questionnaires [18].

**Negative attitude**: respondents who answered agree or strongly agree below the mean from forty-five Likert scale questionnaires [18].

**Evidence-based practice implementation:** Nurses who scored equal and above the median score were categorized as having good evidence-based practice, while those below the Median score as having poor evidence-based practice [7].

**Good Practice**: respondents who answered correctly and those who scored equal to or above the overall mean value from the total of practice-related questions [2].

**Poor Practice**: respondents who answered Practice-related questions below from the mean value of the total of practice-related questions [2].

Note that, good implementation is the score equal to or above the overall median value since the data were not normally distributed, we used the median, and for poor implementation, the score is below the median value. Good practice is a score equal to or above the overall mean value. Since the data were normally distributed, we used the mean value, and poor practice is the score below the mean value.

### Data quality control and management

To ensure the quality of the data, semi-structured data-collecting tools were carefully prepared and pretested in one of the study hospitals called Tora Primary Hospital two weeks before the actual data collection. In the pilot study, internal data consistency (Cronbach’s alpha test) was done on knowledge, attitude, practice, and implementation of EBP, and the results were 0.82, 0.84, and 0.78, respectively. The Cronbach’s alpha evidence-based practice implementation was 0.78. Supervisors and data collectors both received training. The questions were also properly coded and categorized. Each checklist had been used to collect data, and it had been revised, cleaned, and checked for completeness afterward. The principal investigators and supervisors follow the data collection process constantly. Every day following data collection, questionnaires were examined and verified for accuracy. Then, appropriate feedback was offered to data collectors the next morning.

### Data analysis plan and procedures

Epi Data version 4.6.0 was used to originally code and enter the data. For analysis, the data were exported to SPSS version 26 (a statistical tool for social science). Descriptive statistics are used in data analysis to describe the participants’ demographics. Text, tables, graphs, and narration were all used to describe the data. To ascertain the relationship between the outcome variables and predictors, binary logistic regression was performed. Statistical analysis was done with a P-value of 0.05 and a 95% CI. In the multi-variable analysis, variables that had a p-value of up to 0.25 in the bivariate analysis were chosen to fit the model. The Cronbach’s alpha coefficient of 0.78 was used to calculate the internal consistency of outcome indicators. The Hosmer and Lemeshow model’s goodness of fit was tested, and the p-value of 0.341 was greater than the p-value of 0.05. The model summary was also checked by Nagelkerke’s R-squared of 0.225, which was also good. Finally, factors with a statistically significant association with EBP were chosen using a P value of 0.05 or less in the multi-variable logistic regression model.

### Ethics approval and consent to participate

The study was approved by the ethical committee of Saint Paul’s Hospital Millennium Medical College, or the institutional review board (IRB) of Saint Paul’s Hospital Millennium Medical College, with the reference number PM 23/436 on 18/2/23. This letter was written for Siltie Zone Public Hospitals, South Central Ethiopia. 2023.

## Result

### Socio demographic characteristics of nurses in siltie zone public hospitals South central Ethiopia, 2023

From hospitals in the Siltie Zone, 337 nurses in all were found. A total of 323 (95.85%) respondents were capable and willing to participate. Female respondents made up 214 (66.3%) of the total. The majority of respondents, 246 (76.2%), were between the ages of 31 and 35; their median age fell between 31 and 34 years. Among the respondents, 227 (70.3%) were single, and 96 (29.7%) were married. According to the data, 236 (73.1%) currently held bachelor’s degrees in nursing, 71 (22%) had a diploma, and 217 (67.2%) of them had more than five years of work experience. 136 (42.5%) of the total participants worked in inpatient departments. 286 (88.5%) of them were staff nurses (Table 1).

**Table 1:** Socio-demographic Characteristics of Nurses in Siltie Zone Public Hospitals, South Central Ethiopia, 2023 (n = 323).

Variables		Number (%)
Gender	Male	109(33.7%)
	Female	214 (66.3%)
Age	25 and younger	20(6.2%)
	26-30	34(10.5%)
	31-35	246(76.2%)
	36 and older	23(7.12%)
Marital status	Single	227(70.3%)
	Married	96(29.7%)
Educational level	Diploma	71(22%)
	BSc	236(73%)
	Masters and above	16(5%)
Work experience	≤ 5 years	106(32.8%)
	> 5 years	217(67.2%)
Working unit	Emergency	79(24.5%)
	Inpatient	136(42%)
	Outpatient department (OPD)	63(19.4%)
	Maternity and child	16(5%)
	OR	29(9%)
Position	Staff nurse	286(88.5%)
	Head nurse	30 (9.3%)
	Supervisor nurse	3(0.9%)
	Nurse director	4(1.2%)
Working hour	≤ 8 hour	275(85.1%)
	> 8 hour	48(14.9%)

### Individual factors related to evidence-based practice among nurses

#### Evidence-based practice knowledge among nurses.

In evidence-based practice knowledge, 175 (54.2%) of the participants knew how to implement evidence-based practice, and 82 (25.4%) of the nurses identified gaps in their practice. The knowledge of 176 (54.5%) nurses was sufficient. 85 (26.3%) of nurses’ cases heard about evidence-based practice. 56 (17.3%) locate a website that publishes clinically relevant papers, articles, and guidelines. 66 (20.4%) know how to retrieve evidence, but 110 (34.1%) of them had trouble understanding research reports. The overall knowledge of nurses on EBP was 54.5%. The respondent also reported that the reasons for difficulty understanding research reports were 59.48% difficulty in understanding statistical data, 26.72% difficulty in understanding epidemiological terms, and 13.79% difficulty in understanding language. In the implementation of evidence-based practice, knowledge is also linked to educational level, as being less educated undermines evidence-based practice (Table 2)

**Table 2:** Knowledge of evidence-based practice among nurses working in Siltie Zone public Hospitals, South Central Ethiopia, 2023 (n = 323).

Variables	Categories	
	Yes (n (%))	No (n (%))
Have you heard of evidence-based practice or other similar terms?	85(26.3%)	238(73.7%)
Evidence and patients preferences are equally important to make clinical decisions	232(71.8%)	91(28.2%)
Evidence alone is insufficient to make a good clinical decision.	91(%)	232(71.8%)
Within EBP, expert opinion is not considered as a form of evidence.	28(8.7%)	295(91.3%)
Understanding of patient preferences is essential for identifying the best available care.	157(48.6%)	166(51.4%)
Can you name a website that publishes clinical journals, articles, and guidelines?	56(17.3%)	267(82.7%)
Do you have difficulty understanding research reports?	110(34.1%)	231(65.9%)
Do you understand how to apply evidence-based practice effectively enough to change practice?	175(54.2%)	148(45.8%)
Do you identify gaps in your professional practice	82(25.4%)	241(74.6%)
Do you know how to retrieve evidence	66(20.4%)	257(79.6%)

#### Nurses attitude towards evidence-based practice.

Evidence-based practice was seen positively by 136 (42.1%) nurses, and 312 (96.6%) of them agreed that it would enhance the quality of patient care. And 195 (60.4%) of nurses agreed that EBP was relevant to their profession. 192 (59.4%) of them agree that EBP takes much time, and 311 (96.3%) of them agree that EBP training should be given. Time wastage, workload, and individual preference negatively influence EBP (Table 3).

**Table 3:** Attitudes of evidence-based practice among nurses in Siltie zone public hospitals, 2023 (n = 323).

Variables	Categories	
	Agree n(%)	Disagree n(%)
EBP will improve patient care	312(96.6%)	11(3.4%)
EBP is not relevant to nurses	128(39.6%)	195(60.4%)
The value of critical analysis	301(93.2%)	22(6.8%)
Training should be given	311(96.3%)	12(3.7%)
Guidelines improve care	316(97.8%)	7(2.2%)
EBP takes too much time	192(59.4%)	131(40.6%)
Workload is great for EBP	298(92.3%)	25(7.7%)
Clinical practice questioned EBP is a waste of time	173(53.6%)	150(46.4%)
Individual preferred method to EBP implementation	272(84.2%)	51(15.8%)

#### Nurses practice on evidence-based practice.

By the results of the practice-related questions, respondents who answered correctly and those who scored equal or above the overall mean value from the total of practice-related questions were 137(42.4%) of nurses were determined to have good practice, whereas respondents who answered below the overall mean value from the total of practice-related questions were 186 (57.6%) and had poor practices about evidence-based practice. The ability to quickly find the best evidence to address clinical concerns was 160 (49.5%) and 111 (34.4%) implemented research recommendations, and only 100 (31%) confidently judged research reports. (Table 4).

**Table 4:** Practice toward evidence-based practice among nurses in Siltie zone public hospitals, 2023 (n = 323).

Variable	Categories	
	Yes	No
Ability to search best evidence in a time-efficient way	160(49.5%)	163(50.5%)
Formulate a clear question based on a specific patient problem	126(39%)	197(61%)
Access the best resources to implement evidence-based practice	119(36.8%)	204(63.2%)
Implement recommendations of research studies	111(34.4%)	212(65.6%)
Ability to find appropriate research reports	120(37.2%)	203(62.8%)
Confident in judging the quality of research reports	100(31%)	223(69%)

#### Evidence-based practice implementations.

Based on the measurement, the responses from nurses were divided into two groups: those who scored at the median level or higher were seen as having a good evidence-based practice implementation. As a result, 147 (45.5%) have good evidence-based practice implementation. On the other hand, 176 (54.5%) of nurses were found to have poor evidence-based practice.

The majority of nurses, 253 (78.3%), were reported to have patient data and developed clinical questions, and 60 (18.6%) were reported to have looked for pertinent data from recent literature to address clinical questions. 107(32.1%) of nurses never share research evidence with the hospital staff.(Table 5)

**Table 5:** Implementation of evidence-based practice among nurses in siltie zone public hospitals, 2023(n = 323).

Variables	Never	Sometimes	Usually	Often	Always
Collect data and formulate clinical question	253(78.3%)	56(17.3%)	6(1.9%)	5(1.5%)	3(0.9%)
Search relevant evidence from guideline	42(13%)	172(53.4%)	80(24.8%)	18(5.6%)	11(3.4%)
Critically appraised a clinical research	58(18%)	75(23.2%)	114(35.3%)	53(16.4%)	23(7.1%)
Findings clinical literatures	45(13.9%)	137(42.4%)	60(18.6%)	59(18.3%)	22(6.8%)
Use new treatment protocols	69(21.4%)	126(39%)	70(21.7%)	41(12.7%)	17(5.3%)
Share evidence to staffs	216(66.9%)	63(19.5%)	26(8%)	13(4%)	5(1.5%)
Change clinical practice	51(15.8%)	159(49.2%)	81(25.1%)	21(6.1%)	11(3.4%)
Evaluate practice on studies	59(18.3%)	116(35.9%)	100(31%)	36(11.1%)	12(3.7%)
Discussing with patient	55(17%)	137(42.4%)	64(19.8%)	49(15.2%)	18(5.6%)
Integrate expertise evidence	61(18.9%)	141(43.7%)	70(21.7%)	32(9.9%)	19(5.9%)
Integrate patient preference	58(18%)	132(40.9%)	72(22.3%)	48(14.9%)	13(4%)
Evaluate patient outcome	49(15.2%)	162(50.2%)	58(18%)	29(9%)	25(7.7%)

### Characteristics associated with institutions

Nurses were asked questions about institutional and individual factors for evidence-based practice. Accordingly, 222 (68.7%) nurses said that hospital facilities such as computers, internet, and new treatment guidelines were not adequate for evidence-based practice, and 317 (98.1%) nurses reported that lack of training about evidence-based practice made it challenging for them to use the best available data for clinical decision-making. Regarding authority in their workplace, 198 (61.3%) nurses said that a lack of authority makes it difficult to apply evidence-based practice. And 276 (85.4%) nurses said that the unavailability of organizational information (protocols and guidelines) made it difficult to find and apply evidence-based practice, and 270 (82.7%) nurses said that insufficient time to search new treatment protocols and research studies made it difficult to implement evidence-based practice. (Table 6)

**Table 6:** Institutional factors related to evidence-based practice among nurses in Siltie zone public hospitals, 2023(n = 323).

Variables	Categories	
	Agree	Disagree
Inadequate hospital infrastructure	300(92.9%)	23(7.1%)
Lack of training	320(99%)	3(1%)
Troubling Language to use literature	277(85.8%)	46(14.2%)
Patient illiteracy	294(91%)	91(9%)
Low patient awareness make difficult	296(91.6%)	27(8.4%)
Lack of patient education difficult to incorporate patient preference	312(96.6%)	11(3.4%)
Sufficient information	290(89.8%)	33(10.2%)
Lack of regular orientation /training	301(93.2%)	22(6.8%)
Sufficient time to search research	283(87.6%)	40(12.4%)
Lack of authority in the workplace	224(69.3%)	99(30.7%)
Supportive hospital managers for EBP	285(88.2%)	38(11.8%)
Team managers initiate use of EBP	296(91.6%)	27(8.4%)
An absence of interdisciplinary discussion makes it difficult	296(91.6%)	27(91.6%)

#### Factors associated with evidence-based practice implementations.

To identify independent factors that affect the implementation of evidence-based practice, those variables with a p-value of < 0.25 in bivariate analysis were entered into multivariate analysis. Then, a multivariate logistic regression model encompassing all of these variables and variables that had a p-value < 0.05 in a multivariate analysis was significantly associated with evidence-based practice among nurses. Factors associated with the implementation of evidence-based practice were age, educational level, and department/working unit; EBP is not relevant to my profession/attitude, and we need to formulate a clear question based on a specific patient problem/skill.

The age of the nurse and evidence-based practice were highly associated. When compared to nurses aged 36 and older, those between the ages of 26 and 30 had a 5.247 times higher likelihood of good implementation of evidence-based practice (AOR = 5.247, 95% CI: 1.249, 22.047) (p = 0.024).

Evidence-based practice was greatly associated with the work of nurses. When compared to nurses who work in an operating theater, those who work in the emergency department were 3.935 times more likely to have good evidence-based practice (AOR = 3.935, 95% CI: 1.300, 11.914) (p = 0.015). And when compared to nurses working in the operating theater, those working in the inpatient department are 4.187 times more likely to have good implementation of evidence-based practice (AOR = 4.684, 95% CI: 1.633, 13.435) (value = 0.004).

Evidence-based practice was substantially associated with the educational level of nurses. Compared to diploma nurses, BSc nurses were 1.956 times more likely to implement evidence-based practice (AOR = 1.956, 95% CI: 1.035, 3.698) (p = 0.039). And compared to diploma nurses, master’s and above nurses were 4.149 times more likely to have implemented evidence-based practice (AOR = 4.149, 95% CI: 1.088–15.817) (p = 0.037).

When compared to nurses who had a positive attitude/EBP was relevant to their profession, those with a negative attitude were 0.459 times/54.1% less likely to implement evidence-based practice (AOR = 0.459, 95% CI:  .236,.878) (p = 0.019). When compared to nurses who could not formulate a research question, those who could formulate a research question based on a patient problem were 1.844 times more likely to implement evidence-based practice (AOR = 1.844, 95% CI: 1.062, 3.201 (p = 0.03) (Table 7).

**Table 7:** Bivariate and multivariate analyses to identify factors associated with implementation of evidence-based practice among Nurses in Siltie Zone Public Hospitals, 2023 (n = 323).

Variables	Categories	Implementation of EBP		COR (95%CI)	AOR(95%CI)	p-value
		Poor (%)	Good (%)			
Age	25 and younger	7(35%)	13(65%)	6.685(1.731-25.823)	4.093(.813,2.610)	0.080
	26-30	12(35.3%)	22(64.7%)	6.6(1.958-22.246)	5.247(1.249,22.047)*	0.024
	31-35	139(56.5%)	107(43.5%)	2.77(.997-7.703)	2.612(.875,7.790)	0.080
	36 and older	18(78.3%)	5(21.7%)		1	
Department	Emergency	40(50.6%)	39(49.4%)	3.737(1.374-10.169)	3.935(1.300-11.914)*	0.010
	OPD	38(60.3)	25(39.7%)	2.52(.900-7.068)	2.644(.856-8.166)	0.090
	Inpatient	65(47.8%)	71(52.2%)	4.187(1.604-10.929)	4.684(1.633-13.435)*	0.004
	Maternity and Child	10(62.5%)	6(37.5%)	2.3(.594-8.902)	2.149(.488-9.463)	0.310
	OR	23(79.3%)	6(20.7%)		1	
Educational Level	Diploma	48(67.6%)	23(32.4%)		1	
	BSc	124(52.5%)	112(47.5%)	1.885(1.078-3.296)	1.956(1.035-3.698)*	0.039
	≥Masters	4(25%)	12(75%)	6.261(1.819-21.547)	4.149(1.088-15.81)*	0.037
Work experience	5yr &below	48(43.5%)	60(56.6%)	0.513(.321−.821)	0.883(.460-1.697)	0.710
	Above 5 yr	130(59.9%)	87(40.1%)		1	
Can heard EBP	No	137(57.6%)	101(42.4%)	1.6(.972-2.633)	1.031(.580-1.834)	0.910
	Yes	39(45.9%)	46(54.1%)		1	
Practice	Poor	98(52.7%)	88(47.3%)	0.842(.540-1.313)	0.932(.543-1.599)	0.797
	Good	78(56.9%)	59(43.1%)		1	
Attitude	Negative	95(50.8%)	92(49.2%)	0.701(.449-1.096)	1.464(.744-2.879)	0.269
	Positive	81(59.6%)	55(40.4%)		1	
EBP is not relevant to nursing	Yes	82(64.1%)	46(35.9%)	1.915(1.212,3.026)	0.459(.236,.878)	0.019
	No	94(48.2%)	101(51.8%)		1	
Formulate question	Yes	80(63.5%)	46(36.5%)	1.83(1.158-2.892)	1.844(1.062,3.201)*	0.030
	No	96(48.7%)	101(51.3%)		1	
Inadequate Hospital infra-structure	Disagree	9(39.1%)	14(60.9%)	0.512(.215-1.219)	0.529(.195-1.438)	0.210
	Agree	167(55.7%)	133(44.3%)		1	
Patient illiteracy	Disagree	9(31%)	20(69%)	0.342(.151−.777)	0.502(.196-1.288)	0.150
	Agree	167(56.8%)	127(43.2%)		1	
Proficiency of English language	Disagree	19(41.3%)	27(58.7%)	0.538(.286-1.1.013)	0.769(.361-1.636)	0.490
	Agree	157(56.7%)	120(43.3%)		1	
Lack of authority at work place	Disagree	42(42.4%)	57(57.6%)	0.49(.306,.800)	0.787(.440,1.408)	0.420
	Agree	134(59.8%)	90(40.2%)	1		
Absence of interdisciplinary discussion	Disagree	20(74.1%)	7(25.9%)	2.564(1.052-6.247)	1.956(.720-5.316)	0.189
	Agree	156(52.7%)	140(47.3%)		1	

NB * = Significant P-value <0.05 in multivariate and 1=reference category.

## Discussion

The goal of this study was to evaluate the extent to which nurses implement evidence-based practice and associated factors. As per the review of different literature, this is the first study to assess the implementation and factors associated with evidence-based practice among nurses working in Siltie Zone public hospitals, South Central, Ethiopia.

Having that in mind, the current study showed that the current study’s finding was that 54.5% of nurses had sufficient knowledge. It was consistent with a study in Ethiopia, 87% [2] and 95.5% [22]. Inconsistent to study in Nigeria [25], South Africa 32.1% and china36% [26]. The reason for this might be the socio-demographic background of study participants. The reason may also be the country’s educational policies. However, having sufficient knowledge about EBP and its benefits does not necessarily mean that it is implemented and might be related to having up-to-date information about EBP through media, training, or searching the internet. 42.1% of them had a positive attitude toward evidence-based practice implementation, inconsistent with studies in Ethiopia, 55% [2], 64.4% [22], and 73% [18]. The possible explanation, according to these data, is that the majority of hospital staff nurses may have different perspectives and attitudes concerning EBP due to differences in their training, job experience, and degree of certification. Higher-level nurses have a more positive attitude toward evidence-based practice because of their increased expertise. And 42.4% of the participants had excellent skills in evidence-based practice, consistent with a study done in Ethiopia (34%) [22].

The implementations of evidence-based practice among nurses working in the Siltie Zone public hospitals were 45.5%, which is in line with the study finding that was done in the United States of America, 40.8% [27]. This study result was also in line with the study done in Norway on the implementation of evidence-based practice among nurses, which is 40% [28]. Studies in Africa (Nigeria) show that the implementation of EBP by nurses was 30.9% [25] and 34.8% [29]. In Ethiopia, 45.2% [22]. Northwest Ethiopia: 47% [2] and 55% [30]; in Ethiopia, Jimma zone: 16.5% [31]; in Tikur Anbessa Specialized Hospital, Addis Ababa, Ethiopia: 15% [18]; in the southern part of Ethiopia: 57.6% [32].

The multivariate logistic regression showed that the association of independent variables with the implementation of evidence-based practice shows age, educational level, working unit/department, ability to formulate research questions based on patient clinical problems, and attitude concerning the relevance of EBP to the nursing profession were statistically associated with the implementation of evidence-based practice at a p-value < 0.05.

Respondents aged between 26 and 30 had a 5.247 times higher likelihood of good implementation of evidence-based practice (AOR = 5.247, 95% CI: 1.249, 22.047) (p = 0.024), in line with a study done in northern Ethiopia, 25 and 29 years (AOR = 5.98, with a 95% CI 1.34–26.7) [29]. Contrary to the Ethiopian study, however, Tikure Anbessa Specialized Hospital, aged 30–34 years, had a relationship with EBP (AOR: 3.74, 95% CI: 1.2–11.5) [18].

Respondents whose educational level was high (BSc, master’s, and above) were 1.956 times (AOR = 1.956, 95% CI: 1.035, 3.698) (p = 0.039). And 4.149 times (AOR = 4.149, 95% CI: 1.088, 15.817) (p = 0.037) more likely to have implemented evidence-based practice, respectively. This is supported by different literature worldwide. It implies that nurses with higher qualifications than those with lower qualifications were more likely to implement good EBP. This might be because education at the levels of BSc, MSc, and higher tends to be more technically oriented, is improving search methodologies, or is more receptive to the incorporation of EBP into curricula and teaching programs.

Nurses who work in the emergency department and inpatient department were 3.935 times (AOR = 3.935, 95% CI: 1.300, 11.914) (p = 0.015) and 4.187 times (AOR = 4.684, 95% CI: 1.633, 13.435) (p = 0.004) more likely to implement evidence-based practice than nurses who work in OPD (AOR = 2.644, 95% CI:  .856, 8.166, p = 0.091) and maternity and child (AOR = 2.149, 95% CI:  .488, 9.463, p = 0.312). The reason may be the opportunity to get different types of cases and get time to search and implement recent study findings. Nurses working in emergency, there is the need for rapid, high-stakes decision-making and the requirement for standardized, reliable protocols to manage diverse, complex, and unpredictable patient scenarios. For inpatient nurses the EBP focuses on sustained clinical pathways and preventing complications over the course of a hospital stay.

Respondents who could formulate research questions based on patient clinical problems were 1.844 times more likely to implement evidence-based practice (AOR = 1.844, 95% CI: 1.062, 3.201, p = 0.03) compared to those who could not formulate questions. The reason may be that formulating a research question based on a patient problem greatly supports the implementation of evidence-based practice. Nurses who could formulate research questions based on their patients’ clinical problems were good implementations of evidence-based practice. This is supported by [5,7,30,33].

The high proportion of participants aged 31–35 years likely reflects characteristics of the study population. This may be related to the workforce composition of participating hospitals where many nurses recruited during the similar period and remain in active service. In addition, most of participant nurses’ and their families were local resident around that area which made limited staff turnover and spent most of their length of service in those selected hospitals.

### Strength of the study

**The strength of this study was that it was conducted in the rural part of Ethiopia.**

### Limitations of the study

The limitations were the cross-sectional design used simultaneously evaluates variables of the effect of interest and their associated factors, since cross-sectional studies cannot establish a cause-and-effect relationship or analyze behavior over a period of time. For instance, we can prove that education cause better EBP. Since the study was one point in time, we can only indicate it was associated. The other one is self-report data, which is mainly subjective to the honest memory of the participants. A wide confidence interval for ages 26–30 is due to small numbers in the reference group, which made it limited precision. The other limitation is the study cannot be generalized beyond similar rural, public hospital settings and median split without external validation may weaken the main outcome.

## Conclusion

The implementation of evidence-based practice among nurses working in public hospitals in the Siltie zone during March to April 2023 was poor (54.48%). According to the study’s findings, age; educational level; department of employment; the capacity to formulate clinical questions based on patient clinical problems; and the relevance of evidence-based practice to nursing professions all had statistically significant associations with the implementation of evidence-based practice.

## Supporting information

S1 Questionnaire Participant Information Sheet, Consent Form, and the study questionnaire used for data collection. (DOCX)

S1 Check list Completed STROBE Checklist for the study. (DOCX)

## Abbreviations

AOR: adjusted odds ratio
CI: confidence interval
COR: crude odds ratio
EBP: evidence-based practice
ICD: international code of diseases
IQR: interquartile range
IRB: institutional review board
Km: kilometer
OR: odds ratio
PH: primary hospital
South Nation: nationality and peoples region
WCSH: Worabe comprehensive specialized hospital

## References

[1] Zaybak A, Gunes UY, Dikmen Y, Arslan GG. Cultural validation of the Turkish version of evidence-based practice questionnaire. Int J Caring Sci. 2017;10(1):37–46.

[2] Degu AB, Yilma TM, Beshir MA, Inthiran A. Evidence-based practice and its associated factors among point-of-care nurses working at the teaching and specialized hospitals of Northwest Ethiopia: a concurrent study. PLoS One. 2022;17(5):e0267347. doi: 10.1371/journal.pone.0267347 35511810 PMC9070954

[3] Fortney JC, Pyne JM, Burgess JF Jr. Population-level cost-effectiveness of implementing evidence-based practices into routine care. Health Serv Res. 2014;49(6):1832–51. doi: 10.1111/1475-6773.12247 25328029 PMC4254127

[4] Gerrish K, Ashworth P, Lacey A, Bailey J, Cooke J, Kendall S, et al. Factors influencing the development of evidence-based practice: a research tool. J Adv Nurs. 2007;57(3):328–38. doi: 10.1111/j.1365-2648.2006.04112.x 17233652

[5] Shifaza F, Evans D, Bradley H. Nurses’ perceptions of barriers and facilitators to implement EBP in the Maldives. Adv Nurs. 2014;2014(1):698604.

[6] Li S, Cao M, Zhu X. Evidence-based practice: Knowledge, attitudes, implementation, facilitators, and barriers among community nurses-systematic review. Medicine (Baltimore). 2019;98(39):e17209. doi: 10.1097/MD.0000000000017209 31574830 PMC6775415

[7] Alemayehu A, Jevoor PC. Utilisation of evidence-based practice and its associated factors among nurses. Indian J Contin Nurs Educ. 2021;22(2):180–7. doi: 10.4103/ijcn.ijcn_101_20

[8] Boström AM, Rudman A, Ehrenberg A, Gustavsson JP, Wallin L. Factors associated with evidence-based practice among registered nurses in Sweden: a national cross-sectional study. BMC Health Serv Res. 2013;13(1):165. 10.1186/1472-6963-13-165 PMC3648399 23642173

[9] Mashiach Eizenberg M. Implementation of evidence-based nursing practice: nurses’ personal and professional factors? J Adv Nurs. 2011;67(1):33–42. doi: 10.1111/j.1365-2648.2010.05488.x 20969620

[10] Chien L-Y. Evidence-based practice and nursing research. J Nurs Res. 2019;27(4):e29. doi: 10.1097/jnr.0000000000000346 31313747 PMC6641093

[11] Melnyk BM, Gallagher-Ford L, Long LE, Fineout-Overholt E. The establishment of evidence-based practice competencies for practicing registered nurses and advanced practice nurses in real-world clinical settings: proficiencies to improve healthcare quality, reliability, patient outcomes, and costs. Worldviews Evid Based Nurs. 2014;11(1):5–15. doi: 10.1111/wvn.12021 24447399

[12] Pmhnp C, Fineout-overholt E, Stillwell SB, Kathleen M. The seven steps of evidence-based practice. Nurs Clin North Am. 2010;110(1):51–3. 10.1097/01.NAJ.0000366056.06605.d2 20032669

[13] AlibakhshiKenari M, Farzinfard F. Effect of evidence-based education to nursing students on the care skills for patients with mental disorders in the psychiatric ward of martyr Beheshti University of Medical Sciences, Tehran, Iran. Int J Nurs Didact. 2015;5(1):59–61.

[14] Thompson C. Clinical experience as evidence in evidence-based practice. J Adv Nurs. 2003;43(3):230–7. 10.1046/j.1365-2648.2003.02705.x 12859781

[15] Haynes RB, Devereaux PJ, Guyatt GH. Physicians’ and patients’ choices in evidence based practice: evidence does not make decisions, people do. BMJ. 2002;324(7350):1350. 10.1136/bmj.324.7350.1350 PMC1123314 12052789

[16] Murad MH, Montori VM, Guyatt GH. Incorporating patient preferences in evidence-based medicine. JAMA. 2008;300(21):2483–4. 10.1001/jama.2008.730 19050187

[17] Robinson B, Hart AM, Whitney FW. Linking evidence-based nursing practice and patient-centered care through patient preferences. Nurs Adm Q. 2013;37(3):231–41. 10.1097/NAQ.0b013e318295ed6b 23744469

[18] Anbessa T, Hospital S, Ababa A, Hadgu G, Almaz S, Tsehay S. Assessment of Nurses ’ Perceptions and Barriers on Evidence Based Practice in Assessment of Nurses ’ Perceptions and Barriers on Evidence Based Practice in Tikur Anbessa Specialized Hospital Addis Ababa Ethiopia. 2017.

[19] Nuhmani S, Gautham P, Kachanathu S. Plantar fasciitis: a review of literature. Saudi J Sports Med. 2014;14(2):69. doi: 10.4103/1319-6308.142347

[20] Aliyu AS, Yakubu UN, Adamu A, Bura HA, Auwalu Y. Epidemiological assessment of nurses’ knowledge and utilization of evidence based practice and its associated factors in specialist hospital Bauchi state, Nigeria. ARC J Public Health and Commun Medicine. doi: 10.20431/2456-0596.0503004

[21] Khammarnia M, Mohammadi MH, Amani Z, Rezaeian S, Setoodehzadeh F. Barriers to Implementation of Evidence Based Practice in Zahedan Teaching Hospitals, Iran, 2014. 2015;2015. 10.1155/2015/357140 PMC4381851 25866675

[22] Dessie Ayalew G, Jara D, Alem G, Mulugeta H, Zewdu T. Evidence-based practice and associated factors among health care providers working in public hospitals. 2017. 10.1016/j.curtheres.2020.100613 PMC7708748 33306046

[23] Tool CA. Cycle Audit Tool Clinical Service Directorate. 2022.

[24] Emwodew D, Melese T, Takele A, Mesfin N, Tariku B. Knowledge and attitude toward evidence-based medicine and associated factors among medical interns in amhara regional state teaching hospitals, northwest ethiopia: cross-sectional study. JMIR Med Educ. 2021;7(2). 10.2196/28739 PMC8277356 34185012

[25] Oluwatoyin FE, EkeFJH BF. Knowledge and utilization of evidence-based nursing practice among nurses of Offa Specialist Hospital, Kwara State. IOSR J Nurs Health Sci. 2016;4:51–62.

[26] Weng Y-H, Chen C, Kuo KN, Yang C-Y, Lo H-L, Chen K-H, et al. Implementation of evidence-based practice in relation to a clinical nursing ladder system: a national survey in Taiwan. Worldviews Evid Based Nurs. 2015;12(1):22–30. doi: 10.1111/wvn.12076 25588625 PMC4345401

[27] Harper MG, Gallagher-Ford L, Warren JI, Troseth M, Sinnott LT, Thomas BK. Evidence-based practice and US healthcare outcomes: findings from a national survey with nursing professional development practitioners. J Nurses Prof Dev. 2017;33(4):170–9. 10.1097/NND.0000000000000360 28441160

[28] Stokke K, Olsen NR, Espehaug B, Nortvedt MW. Evidence based practice beliefs and implementation among nurses: a cross-sectional study. BMC Nurs. 2014;13(1):8. doi: 10.1186/1472-6955-13-8 24661602 PMC3987836

[29] Dagne AH, Beshah MH, Kassa BG, Dagnaw EH. Implementation of evidence-based practice and associated factors among nurses and midwives working in Amhara Region government hospitals: a cross-sectional study. Reprod Health. 2021;18(1):36. doi: 10.1186/s12978-021-01096-w 33579309 PMC7881559

[30] Aynalem ZB, Yazew KG, Gebrie MH. Evidence-based practice utilization and associated factors among nurses working in Amhara Region Referral Hospitals, Ethiopia. PLoS One. 2021;16(3):e0248834. doi: 10.1371/journal.pone.0248834 33740000 PMC7978364

[31] Dereje B, Hailu E, Beharu M. Evidence-based practice utilization and associated factors among nurses working in public hospitals of Jimma zone southwest Ethiopia: a cross sectional study. Gen Med (Los Angel). 2019;7(1):321.

[32] Tadesse B, Hw T, Ad E. Assessment of nurses knowledge and utilization of evidence based practice and its associated factors in selected hospitals of southern Ethiopia.

[33] Worku T, Yeshitila M, Feto T, Leta S, Mesfin F, Mezmur H. Evidence-based medicine among physicians working in selected public hospitals in eastern Ethiopia: a cross-sectional study. BMC Med Inform Decis Mak. 2019;19(1). 10.1186/s12911-019-0826-8 PMC6547595 31159784

