# Supplementary material for: Evidence-based practice implementation and associated factors among nurses employed at public hospitals in Siltie Zone, South Central Ethiopia: A cross-sectional study
Source: PLoS One. 2026 Oct 20;21(10):e0357626. doi: 10.1371/journal.pone.0357626 (PMC13682656; doi:10.1371/journal.pone.0357626)
Supplement: Supplementary file 1 [file pone.0357626.s001.docx]

1. **Annexes**

**Annex 1: Participant Information Sheet and Consent Form**

Saint Paul’s Hospital Millennium medical College, School of Nursing Graduate Studies

Hello! My name is……………. I am here on behalf of Sadik Seid student of Saint Paul’s Hospital Millennium medical College, School of Nursing. He is conducting a research for the Partial fulfillment of master’s degree on ‘evidence-based practice implementation and associated factors among nurses working in public hospitals of Silte Zone, South central Ethiopian’. The information you provide will help to indicate potential intervention points for stakeholders by assessing implementation of evidence-based practice and identifying associated factors of evidence-based practice. Your name will not be recorded in any part of the questionnaire in order to ensure confidentiality of the information you provide. The study does not have any harm to the study nurses. This questionnaire will take only 25-30 minutes. You may ask us to clarify questions if you do not understand or can stop the interview at any time. For any questions, you can contact the principal investigator Sadik Seid by using phone number +251912903037.

Are you willing to participate in this study? No (thank you) Yes (continue with the interview)

Signature for consent____________

Name of Hospital_________________________

Date of interview (Ethiopian calendar) _____|_____| ______| Start time____ End time_____

Questionnaire with ID number of study subjects (to be numbered before interview)

ID Number of nurse _______________

**Annex II: Questionnaires**

**Part I. Research Questionnaire checklist (adapted from related literature review).**

| Part A:Basic socio-demographic characteristics of the respondents | | | | | | | | | | | | | | | | | | | | | |
| --- | --- | --- | --- | --- | --- | --- | --- | --- | --- | --- | --- | --- | --- | --- | --- | --- | --- | --- | --- | --- | --- |
| Q.no. | | Questions | | | | | Choice for response | | | | | | | | | | | | Skip | | |
| A01 | | Age | | | | | Age in year _____ | | | | | | | | | | | |  | | |
| A02 | | Gender | | | | | 1. Male 2. Female | | | | | | | | | | | |  | | |
| A03 | | What is your current marital status? | | | | | 1. Single 2. Married 3. Divorced 4. Widowed | | | | | | | | | | | |  | | |
| A04 | | What is your current educational level? | | | | | 1. Diploma nurse 2. BSc nurse 3. Master and above nurse | | | | | | | | | | | |  | | |
| A05 | | Tell me your work experience | | | | | _________years | | | | | | | | | | | |  | | |
| A06 | | In what department you work? | | | | | 1. Emergency departments 2. OPD departments 3. Inpatient departments 4. Maternity and child 5. Operating theaters 6. Antiretroviral therapy 7. Other (specify)_______ | | | | | | | | | | | |  | | |
| A07 | | What is your current position? | | | | | 1. Staff nurse 2. Head nurse 3. Supervisor nurse 4. Nurse director 5. Other specify________ | | | | | | | | | | | |  | | |
| A08 | | How long you work per day? | | | | | In hours_______ | | | | | | | | | | | |  | | |
| Part B: questions related to knowledge about evidence-based practice. | | | | | | | | | | | | | | | | | | | | | |
| B01 | | Have you heard of evidence-based practice or other similar terms? | | | | 1. Yes 2. No | | | | | | | | | | | | | |  | |
| B02 | | If Yes for QB01 what you know about EBP? | | | | explain------------------------------ | | | | | | | | | | | | | |  | |
| B03 | | Evidence and patients are equally important to make clinical decisions. | | | | 1. Yes 2. No | | | | | | | | | | | | | |  | |
| B04 | | Evidence alone is insufficient to make a good clinical decision. | | | | 1. Yes 2. No | | | | | | | | | | | | | |  | |
| B05 | | Within EBP, expert opinion is not considered as a form of evidence. | | | | 1. Yes 2. No | | | | | | | | | | | | | |  | |
| B06 | | Understanding of patient preferences is essential for identifying the best available care. | | | | 1. Yes 2. No | | | | | | | | | | | | | |  | |
| B07 | | Can you identify a website that publishes clinical journals, articles, and guidelines? | | | | 1. Yes 2. No | | | | | | | | | | | | | |  | |
| B08 | | Do you have difficulty understanding research reports? | | | | 1. Yes 2. No | | | | | | | | | | | | | |  | |
| B09 | | If yes for Q. No B03 What is the reason for difficulty? | | | | 1. Understanding statistical data 2. Understanding epidemiological terms 3. Language problems | | | | | | | | | | | | | |  | |
| B10 | | Do you understand how to apply evidence-based practice effectively enough to change practice? | | | | 1. Yes 2. No | | | | | | | | | | | | | |  | |
| B11 | | Do you identify gaps in your professional practice | | | | 1. Yes 2. No | | | | | | | | | | | | | |  | |
| B12 | | Do you know how to retrieve evidence | | | | 1. Yes 2. No | | | | | | | | | | | | | |  | |
| Part C: questions related to attitudes about evidence-based practice(tick on your choice) | | | | | | | | | | | | | | | | | | | | | |
|  | | | | strongly  disagree | | | | | | | | disagree | | neutral | | agree | | | | strongly agree | |
| C01 | | Implementing EBP will improve the care that I deliver to my patients | |  | | | | | | | |  | |  | |  | | | |  | |
| C02 | | EBP is not relevant to my profession | |  | | | | | | | |  | |  | |  | | | |  | |
| C03 | | Critically appraising evidence is an important step in the EBP process | |  | | | | | | | |  | |  | |  | | | |  | |
| C04 | | Training should be given about EBP | |  | | | | | | | |  | |  | |  | | | |  | |
| C05 | | Recent national guidelines improve clinical care | |  | | | | | | | |  | |  | |  | | | |  | |
| C06 | | EBP takes too much time so it is difficult to implement | |  | | | | | | | |  | |  | |  | | | |  | |
| C07 | | My workload is too great for me to keep up-to-date with all the new evidence | |  | | | | | | | |  | |  | |  | | | |  | |
| C08 | | I resent having my clinical practice questioned evidence-based practice is a waste of time | |  | | | | | | | |  | |  | |  | | | |  | |
| C09 | | I stick to tried and trusted methods rather than changing to anything new | |  | | | | | | | |  | |  | |  | | | |  | |
| Part D: questions related to skill of evidence-based practice. | | | | | | | | | | | | | | | | | | | | | |
| D01 | Can you search for the best evidence to answer clinical questions in a time-efficient way? | | | | | | | | | | 1. Yes 2. No | | | | | | | | |  | |
| D02 | If yes for Q D01from what source you search? | | | | | | | | | | Specify it_____________ | | | | | | | | |  | |
| D03 | Can you formulate a clear question based on a specific patient problem? | | | | | | | | | | 1. Yes 2. No | | | | | | | | |  | |
| D04 | Can you access the best resources to implement evidence-based practice? | | | | | | | | | | 1. Yes 2. No | | | | | | | | |  | |
| D05 | Do you have the ability to implement recommendations of research studies into clinical practice? | | | | | | | | | | 1. Yes 2. No | | | | | | | | |  | |
| D06 | Do you have the ability to find appropriate research reports? | | | | | | | | | | 1. Yes 2. No | | | | | | | | |  | |
| D07 | Do you feel confident in judging the quality of research reports? | | | | | | | | | | 1. Yes 2. No | | | | | | | | |  | |
| Part E:questions related to status of evidence based practice implementation(tick on your choice) | | | | | | | | | | | | | | | | | | | | | |
|  |  | | | | Never | | | Sometimes | | | | | | | Usually | | | Often | | | Always |
| E1 | Collect data and formulate clinical question | | | |  | | |  | | | | | | |  | | |  | | |  |
| E2 | Search relevant evidence from recent national guidelines and literatures | | | |  | | |  | | | | | | |  | | |  | | |  |
| E3 | Read and critically appraised a clinical research study | | | |  | | |  | | | | | | |  | | |  | | |  |
| E4 | Apply the findings of clinical literatures into clinical practice | | | |  | | |  | | | | | | |  | | |  | | |  |
| E5 | Use recent national guidelines, new treatment protocols to give care | | | |  | | |  | | | | | | |  | | |  | | |  |
| E6 | Share evidence from studies in the form of report or presentation to hospital staffs | | | |  | | |  | | | | | | |  | | |  | | |  |
| E7 | Change existed clinical practice based on recent clinical studies | | | |  | | |  | | | | | | |  | | |  | | |  |
| E8 | Evaluate practice based on recent studies | | | |  | | |  | | | | | | |  | | |  | | |  |
| E9 | Discussing with patient/family member to change new types of care | | | |  | | |  | | | | | | |  | | |  | | |  |
| E10 | Integrate the evidence found with expertise | | | |  | | |  | | | | | | |  | | |  | | |  |
| E11 | Integrate patient preference in practice | | | |  | | |  | | | | | | |  | | |  | | |  |
| E12 | Evaluate the patient outcome after practice change | | | |  | | |  | | | | | | |  | | |  | | |  |
| Part F: question related to factors to evidence-based practice (tick on your choice) | | | | | | | | | | | | | | | | | | | | | |
|  | | | strongly disagree | | | | | | disagree | | | | neutral | | | | agree | | | strongly agree | |
| F01 | Hospital infrastructure, such as computers, Internet, and new treatment guidelines, is not adequate for implementation of EBP | |  | | | | | |  | | | |  | | | |  | | |  | |
| F02 | Lack of training about EBP makes it difficult to implement | |  | | | | | |  | | | |  | | | |  | | |  | |
| F03 | Problems understanding English makes it difficult to use the literature | |  | | | | | |  | | | |  | | | |  | | |  | |
| F04 | Patient illiteracy makes it difficult to discuss information regarding management | |  | | | | | |  | | | |  | | | |  | | |  | |
| F05 | Low patient awareness about their disease management makes it difficult to implement EBP | |  | | | | | | |  | | |  | | | |  | | |  | |
| F06 | Lack of an organized patient education department makes it difficult to incorporate patient preferences into practice | |  | | | | | | |  | | |  | | | |  | | |  | |
| F07 | There is sufficient information to find new guidelines/protocols | |  | | | | | | |  | | |  | | | |  | | |  | |
| F08 | There is a lack of regular orientation /training about new health priority issues | |  | | | | | | |  | | |  | | | |  | | |  | |
| F09 | There is sufficient time to find new guidelines/protocols or online research findings | |  | | | | | | |  | | |  | | | |  | | |  | |
| F10 | There is a lack of authority in the workplace to change practice | |  | | | | | | |  | | |  | | | |  | | |  | |
| F11 | Hospital managers are supportive of EBP | |  | | | | | | |  | | |  | | | |  | | |  | |
| F12 | Team managers initiate the use of EBP | |  | | | | | | |  | | |  | | | |  | | |  | |
| F13 | An absence of interdisciplinary discussion during patient management makes it difficult to apply evidence to practice | |  | | | | | | |  | | |  | | | |  | | |  | |

Name of data collector __________________________	signature__________

Thank you!
